# Effect of Stretching on Crystalline Structure, Ferroelectric and Piezoelectric Properties of Solution-Cast Nylon-11 Films

**DOI:** 10.3390/polym13132037

**Published:** 2021-06-22

**Authors:** Jima Wu, Yuheng Fu, Guo-Hua Hu, Shan Wang, Chuanxi Xiong

**Affiliations:** 1 School of Materials Science and Engineering, Wuhan University of Technology, Wuhan 430070, China; jmw-278361@whut.edu.cn (J.W.); yuhengfu2021@163.com (Y.F.); 2 Université de Lorraine-CNRS, Laboratory of Reactions and Process Engineering (LRGP, UMR CNRS 7274), 1 rue Grandville, BP 20451, 54001 Nancy, France; guo-hua.hu@univ-lorraine.fr

**Keywords:** nylon-11, stretching, crystalline structure, ferroelectricity, piezoelectricity

## Abstract

Compared to polyvinylidene fluoride (PVDF) and its copolymers, castor-oil-derived nylon-11 has been less explored over the past decades, despite its excellent piezoelectric properties at elevated temperatures. To utilize nylon-11 for future sensor or vibrational energy harvesting devices, it is important to control the formation of the electroactive δ′ crystal phase. In this work, nylon-11 films were first fabricated by solution-casting and were then uniaxially stretched at different stretching ratios (SR) and temperatures (T_s_) to obtain a series of stretched films. The combination of two-dimensional wide-angle X-ray diffraction (2D-WAXD) and differential scanning calorimetry (DSC) techniques showed that the fraction of the δ′ crystal phase increased with the stretching ratio and acquired a maximum at a T_s_ of 80 °C. Further, it was found that the ferroelectric and piezoelectric properties of the fabricated nylon-11 films could be correlated well with their crystalline structure. Consequently, the stretched nylon-11 film stretched at an SR of 300% and a T_s_ of 80 °C showed maximum remanent polarization and a remarkable piezoelectric coefficient of 7.2 pC/N. A simple piezoelectric device with such a nylon-11 film was made into a simple piezoelectric device, which could generate an output voltage of 1.5 V and a current of 11 nA, respectively.

## 1. Introduction

Piezoelectric materials having non-centrosymmetric point groups can develop net deformation from their equilibrium positions with the application of an electric field or mechanical stress, consequently allowing for the conversion of a mechanical stress into an electrical charge or an electrical field into a mechanical strain, known as the “direct” effect and the “converse” effect, respectively [1]. By utilizing the direct or converse piezoelectric effect, nowadays piezoelectric materials have been widely designed into various smart devices. For example, pressure sensors, wearable electronic textiles and vibrational energy harvesting devices utilize the direct piezoelectric effect, while the shape control and movement actuation count on the converse piezoelectric effect [2,3,4,5,6,7,8].

Since the discovery of piezoelectricity in polyvinylidene fluoride (PVDF) by Kawai in 1969, the piezoelectric effect has also been found in other polymers, including PVDF copolymers, odd nylons, cellulose and poly (lactic acid) [9,10,11,12]. Although they have a relatively lower piezoelectric coefficient than their ceramic counterparts, piezoelectric polymers typically provide flexibility and processability, allowing integration into small, lightweight devices for applications such as human body movement monitoring and human-based or low-frequency ambient energy harvesting [13,14].

Among piezoelectric polymers, a castor-oil-derived nylon-11 has been less explored over the past decades, although it possesses the advantage δ’ of despite its excellent piezoelectric properties at elevated temperatures compared to PVDF and its copolymers [15]. It is now well recognized that nylon-11 typically owns polymorphic crystal forms, namely α, α′, γ and δ’, of which δ’-form can respond well to the applied an electric field and thus show superior ferroelectric and piezoelectric properties [10,16,17]. Therefore, achieving nylon-11 films with a large fraction of δ’-form has been highly pursued during the past years [18]. Usually, nylon-11 is crystallized from the molten state into the triclinic α- or α′-form, which does not exhibit piezoelectricity due to the random polarity of the crystalline domains that cannot be rearranged by electric poling because of the strong interchain hydrogen bonding [19,20]. Earlier research on solution-cast nylon-11 films also showed that non-piezoelectric α-forms are commonly generated when employing a phenol/formic acid mixture as the solvent [19]. According to previous studies, δ’-form nylon-11 can only be attained by quenching the melt at ultrafast cooling speeds or drawing the films at low temperatures [21,22]. Recently, a few investigations have attempted to achieve ferroelectric or piezoelectric nylon-11 films via selecting suitable solvents [23,24,25]. For instance, Anwar et al. reported a transparent ferroelectric nylon-11 thin film processed from a trifluoroacetic acid (TFA)/acetone mixture [26]. However, researches on understanding of the crystalline structure of solution-cast nylon-11 films are still quite limited, and especially its the crystalline structural evolution upon post-treatment like uniaxial stretching has been less focused on.

In this study, a nylon-11 film was fabricated by solution casting from a mixture of formic acid (FA) and dichloromethane (DCM). The crystalline structural evolution (crystalline form, orientation and crystallinity) upon post-stretching was comprehensively investigated by the two-dimensional wide-angle X-ray diffraction (2D-WAXD) method, as well as differential scanning calorimetry (DSC). Then, the ferroelectric and piezoelectric properties were studied and well-correlated with their crystalline structure. Finally, simple piezoelectric devices based on those nylon-11 films were fabricated, and their output voltage and current responding to an applied force were comparatively evaluated.

## 2. Materials and Methods

### 2.1. Materials and Sample Preparation

Nylon-11 (Rilsan^®^BESNO TL) was purchased from Akema-Rilsan Co. Ltd., Paris, France. Formic acid (reagent grade ≥ 95%) and dichloromethane (reagent grade ≥ 98%) were bought from Sinopharm Group, Shanghai, China.

An unstretched nylon-11 film was prepared by the solution casting method. Typically, 10 wt% of nylon-11 solution was first prepared by dissolving the nylon-11 pellets into a mixture of formic acid and dichloromethane (1: 2 volume ratio) at room temperature. The as-made nylon-11 solution was then cast onto a glass substrate placed on a hot plate. After being heated at 80 °C for 2 h, the sample was transferred into a vacuum oven at 80 °C for 24 h to evaporate the residual solvent. To obtain a series of stretched films, the as-prepared film with a thickness of around 30 μm was uniaxially extended at different stretching ratios (i.e., 100%, 200% or 300%) and temperatures (i.e., 25 °C, 80 °C, 120 °C or 145 °C). A stretching ratio of 300% was adopted as the upper limit in order to avoid film rupture since the risk of film rupture would increase when exceeding this value. All the stretching processes were carried out using a universal tensile testing machine (SANS CMT6503, Shenzhen, China) at a strain rate of 10 mm/min.

### 2.2. Characterizations

To determine the crystalline structural evolution upon stretching, the two-dimensional wide-angle X-ray diffraction (2D-WAXD) patterns of the stretched and unstretched nylon-11 films were recorded by XeUSS2.0 (Xenocs, Sassenage, France) using Cu Kα radiation (λ = 0.154 nm). The operating voltage and current were 50 kV and 0.6 mA, respectively.

The melting behavior of the stretched and unstretched nylon-11 films was examined using differential scanning calorimetry (DSC) by a Q25 Instrument (TA instrument, New Castle, DE, USA). Each sample was heated from room temperature to 200 °C in nitrogen atmosphere at a heating rate of 10 °C min^−1^. The degree of crystallinity (*X*_c_) was calculated as $X_{c}=\frac{∆H}{∆H_{m}^{0}}$, where ∆H and $∆H_{m}^{0}$ are the fusion enthalpy of the sample and 100% crystalline nylon-11 (206 J g^−1^), respectively [27].

The displacement-electric field hysteresis loop of each film was measured using a modified Sawyer–Tower ferroelectric testing system (PolyK, North Philipsburg, PA, USA) with a bipolar sinusoidal wave of 1 Hz. Before the measurement, gold electrodes having a thickness of 50 nm and a diameter of 10 mm were sputtered on both sides of the film by a GVC-1000 ion sputtering instrument (Beijing Gevee-Tech Co. Ltd., Beijing, China).

The piezoelectric coefficient (d_33_) was measured by a quasi-state d_33_ piezometer (ZJ-3AN, Beijing, China). The frequency and amplitude of the applied force were 110 Hz and 0.25 N, respectively. All the samples were thermally poled at 80 °C for 30 min under an electric field of 100 MV/m before measurement.

The open-circuit voltage and short-circuit current were measured by an electrometer (KEITHLEY 6514, Solon, OH, USA). The applied force and its frequency were controlled by a stepping motor (SBT951-T, Simbatouch, Guangzhou, China). Before testing, both sides of the film were attached to aluminum electrodes and then encapsulated with polyimide films.

## 3. Results and Discussion

### 3.1. Effect of Stretching on Crystalline Structure of Nylon-11 Film

**Figure 1:**
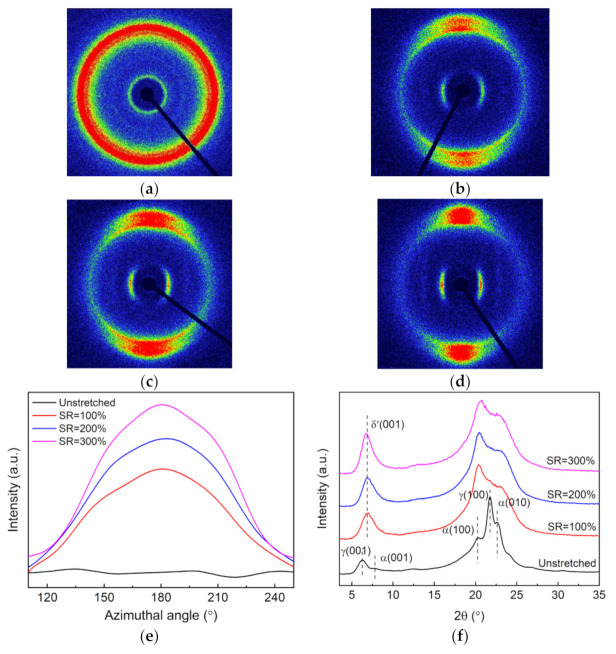
2D-WAXD patterns of the nylon-11 films: (**a**) unstretched and (**b**–**d**) stretched at 80 °C with different stretching ratios (SR): (**b**) 100%, (**c**) 200% and (**d**) 300%; (**e**) azimuthal intensity distributions of the (001) reflection, and (**f**) diffraction intensity profiles derived from the 2D-WAXD patterns.

The crystalline structural evolution of the nylon-11 film upon stretching at 80 °C with different ratios was studied by the 2D-WAXD technique and the results are shown in Figure 1. As seen from Figure 1a, the 2D-WAXD pattern of the unstretched nylon-11 film exhibits a collection of several concentric rings, suggesting an isotropic crystalline structure. As the stretching ratio (SR) increases, these concentric rings turn into arcs and their length gradually decreases. The diffraction arc of the (001) crystalline plane (the innermost circle) progressively converges towards the equator direction, while those of the (100) and (010/110) planes (the outer circles) gradually converge towards the meridian direction (Figure 1a–d). The result suggests that the crystalline domains of the nylon-11 would be orientationally aligned by applying the tensile stress and that the orientation increases when increasing the SR. The azimuthal intensity distributions of the (001) reflection in Figure 1e further confirm that the crystalline orientation increases with the increase of the SR. Figure 1f depicts the diffraction intensity profiles of the nylon-11 films derived from the 2D-WAXD patterns. Distinct differences can be observed among the diffraction intensity curves of the unstretched and stretched nylon-11 films. Specifically, at a low 2θ range of less than 10°, the unstretched nylon-11 film displays two diffraction peaks at 6.2° and 7.8° corresponding to the α and γ phases, respectively, while all the stretched films show only one diffraction peak at 6.8° belonging to the δ’ phase [28]. Besides, a distinct diffraction peak of the γ phase appears at 21.8° for the unstretched nylon-11 film, while it disappears for these stretched films [29].

**Figure 2:**
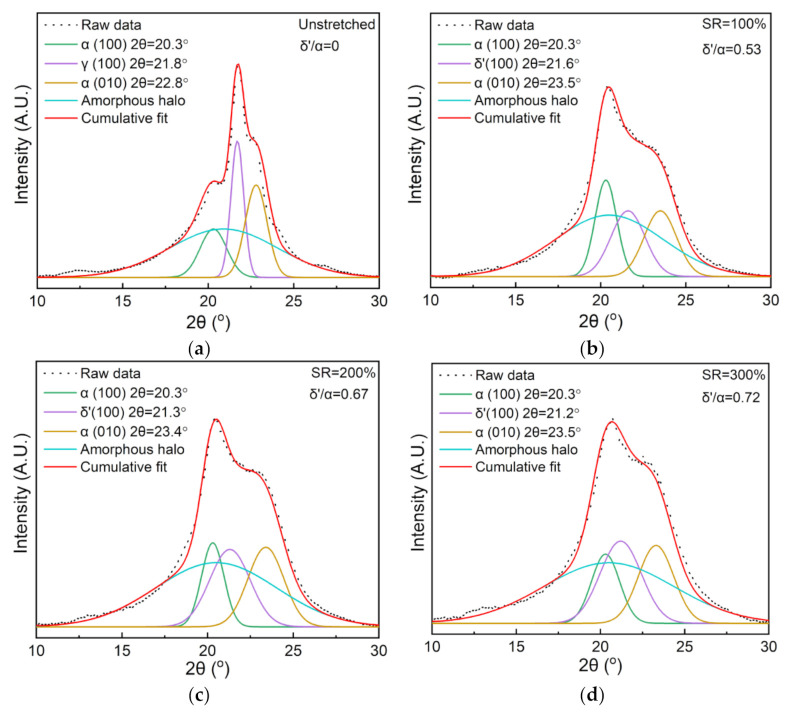
XRD fitting curves for the diffraction peaks of the unstretched and stretched nylon-11 films: (**a**) unstretched; (**b**) SR = 100%; (**c**) SR = 200%; (**d**) SR = 300%.

To further clarify the changes in the crystalline structure, XRD peak fitting in the 2θ range of 10° to 30° was performed and the results are shown in Figure 2. It is found that stretching causes the γ phase to disappear and leads to the generation of the δ’ phase in the nylon-11 films. The fraction ratio of the δ’ phase to the α phase (δ’/α) is calculated as increases from 0.53 to 0.72 as the SRs increase from 100% to 300%. Moreover, the diffraction angle of the (100) plane of the δ’ phase decreases with the increase of the SR, suggesting that the interplanar spacing gets broader widens upon stretching.

**Figure 3:**
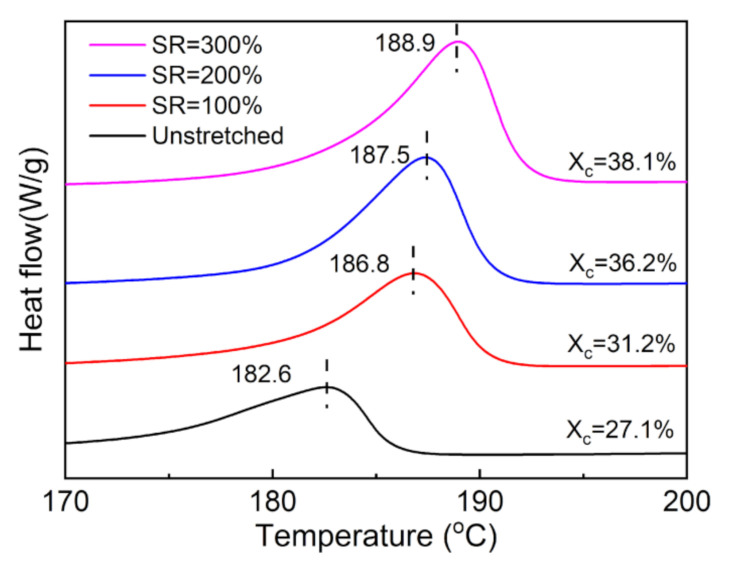
DSC melting curves of the nylon-11 films unstretched and stretched at 80 °C with different stretching ratios (SRs).

Figure 3 presents the DSC melting curves of the unstretched and stretched nylon-11 films at 80 °C with different SRs. The unstretched nylon-11 film displays a relatively low melting point (T_m_) of around 182.6 °C, which is less than the value (186 °C) given by the supplier [30]. This is probably due to the plasticization effect of some residual trace of the formic acid in the nylon-11 film [31]. Stretching shifts the T_m_ to higher temperatures, which can be ascribed to the exclusion of the plasticizer upon stretching. Moreover, increasing the SR causes a significant improvement in the crystallinity of the nylon-11 films. The crystallinity rises from 27.1% for the unstretched nylon-11 film to 38.1% for the 300% stretched film, which is enhanced by nearly 40%. Combined with the previous XRD analysis, the increase in the crystalline fraction could provide the nylon-11 films with more opportunity to achieve a high ferroelectric and piezoelectric performance.

**Figure 4:**
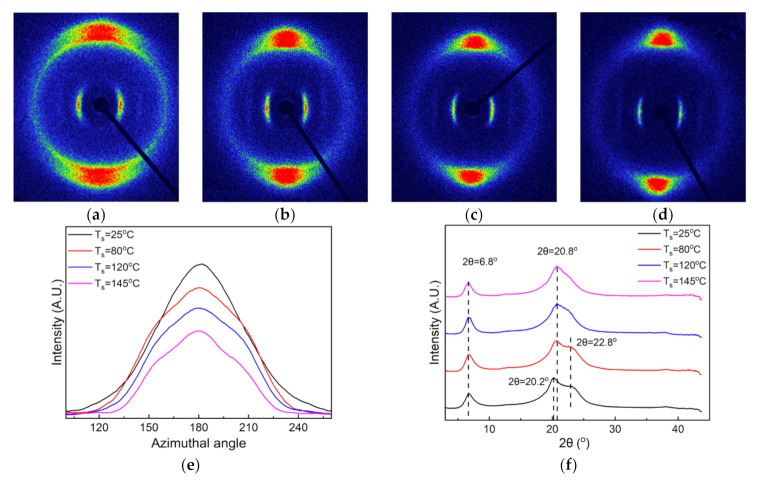
(**a**–**d**) 2D-WAXD patterns of the nylon-11 films stretched at different temperatures: (**a**) 25 °C, (**b**) 80 °C, (**c**) 120 °C and (**d**) 145 °C; (**e**) azimuthal intensity distributions of the (001) reflection, and (**f**) diffraction intensity profiles derived from the 2D-WAXD patterns.

**Figure 5:**
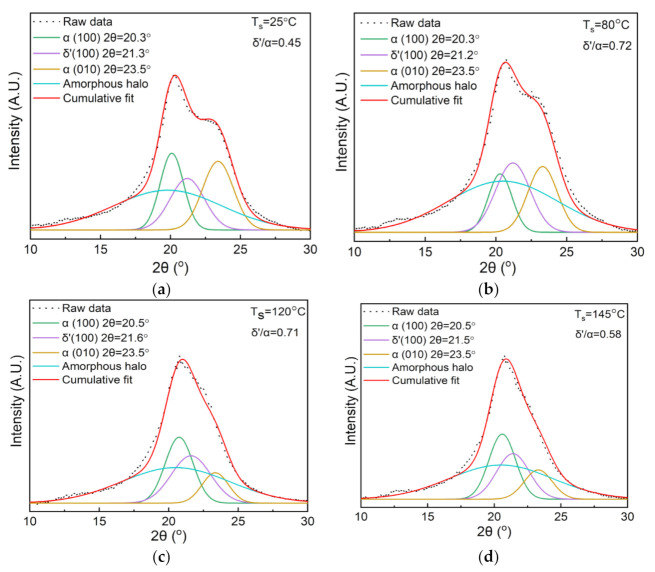
XRD fitting curves for the diffraction peaks of the stretched nylon-11 films with different temperatures. (**a**) 25 °C; (**b**) 80 °C; (**c**) 120 °C; (**d**) 145 °C.

The crystalline structural evolution of the nylon-11 film upon stretching (SR = 300%) at different temperatures was also studied by the 2D-WAXD technique, as shown in Figure 4. As seen from Figure 4a–d, the diffraction arc of the (001) crystalline plane (the innermost ring) progressively converges towards the equator direction, while those of the (100) and (010/110) planes (the outer rings) gradually converge towards the meridian direction when increasing the stretching temperature. The result suggests that increasing the stretching temperature in the range of this study is beneficial to the orientational alignment of the crystalline domains of the nylon-11. The azimuthal intensity distributions of the (001) reflection in Figure 4e further confirm that the crystalline orientation increases with the increased stretching temperature. Figure 4f depicts the diffraction intensity profiles of the nylon-11 films derived from the 2D-WAXD patterns. Changes can be observed among these diffraction intensity curves, especially in the 2θ range of 10° to 30°. For clarity, XRD peak fitting was performed, as shown in Figure 5a–d. The calculated fraction ratio of the δ′ phase to α phase (δ′/α) first increases from 0.45 to 0.72 as the stretching temperature increases from 25 °C to 80 °C. However, when the stretching temperature is higher than 120 °C, a significant decline can be observed in the value of the δ′/α, which finally reaches 0.58 at 145 °C. The result suggests that an overly high stretching temperature may have an adverse effect on the generation of the δ′ phase, which is consistent with the previous study by Zhang et al. [16].

**Figure 6:**
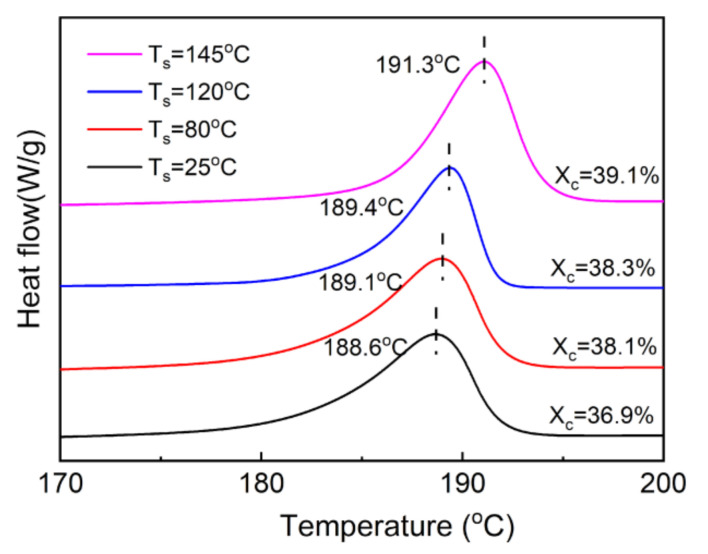
DSC melting curves of the stretched nylon-11 films with different stretching temperatures (T_s_). The stretching ratio is set as 300%.

The DSC melting curves of the 300% stretched nylon-11 films at different stretching temperatures are shown in Figure 6. It is observed that increasing the stretching temperature causes noticeable rises in increases both the melting point and crystallinity. Notably, stretching the nylon-11 film at 145 °C increases the melting point to 191.3 °C, a value that is improved by nearly 9 °C compared to that of the unstretched film. The result is reasonable since a higher temperature enables the molecular chains to have more mobility, and consequently they rearrange themselves more easily into the crystalline region.

### 3.2. Effect of Stretching on Ferroelectric and Piezoelectric Properties of nylon-11 Film

**Figure 7:**
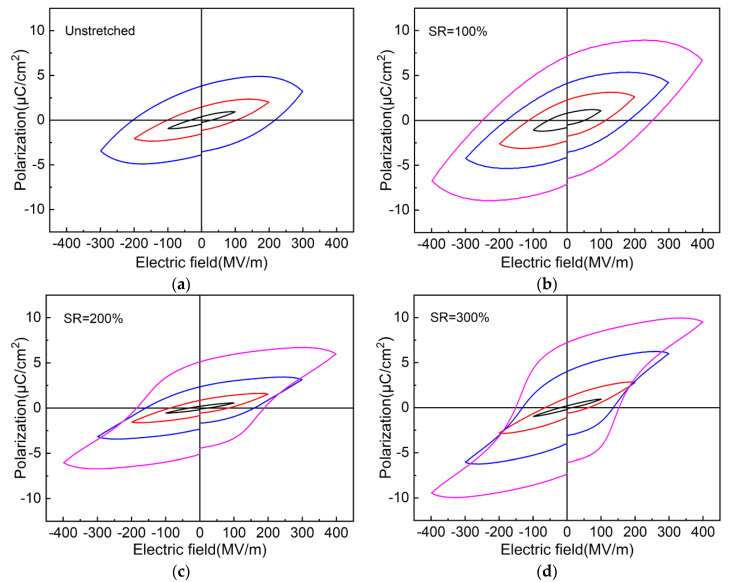
*D*-*E* hysteresis loops of the unstretched and stretched nylon-11 films with different stretching ratios (SRs): (**a**) original, (**b**) 100%, (**c**) 200% and (**d**) 300%. The stretching temperature is 80 °C.

Figure 7a–d show the *D*-*E* hysteresis loops of the unstretched and stretched nylon-11 films at different electric fields, respectively. It should be noted here that the maximum loadable field strength of the unstretched nylon-11 film is about 300 MV/m or so, whereas those of the stretched films can attain 400 MV/m. For all the samples, the polarization hysteresis loops become fatter as the electric field increases. However, the polarization behavior of both the unstretched and 100% stretched nylon-11 films (Figure 7a,b) does not exhibit typical ferroelectric hysteresis loops even at their respective maximum field strength. When the SR is up to 200% or more, typical ferroelectric hysteresis loops can be seen (Figure 7c,d) when the nylon-11 films are subjected to triangular electric fields of more than 300 MV/m. The 300% stretched nylon-11 film exhibits a broader hysteresis loop than the 200% stretched film under the same electric field. Specifically, the remnant polarization (the intercept between the hysteresis loop and *y*-axis) increases from 5.2 μC/cm^2^ to 7.2 μC/cm^2^ as the stretching ratio increases from 200% to 300%, whereas the coercive field (the intercept between the hysteresis loop and *x*-axis) decreases from 187 MV/m to 153 MV/m. The result suggests that stretching nylon-11 film with larger SRs causes more ferroelectric crystalline domains to reverse more easily under the applied electric field. This is reasonable since the XRD analysis in Figure 2 has shown that higher fractions of the ferroelectric δ′ crystalline phases are generated in the stretched nylon-11 film with larger SRs, which can thus respond well to the applied electric field due to their sparse and weak hydrogen bonding in the crystalline domains [25].

**Figure 8:**
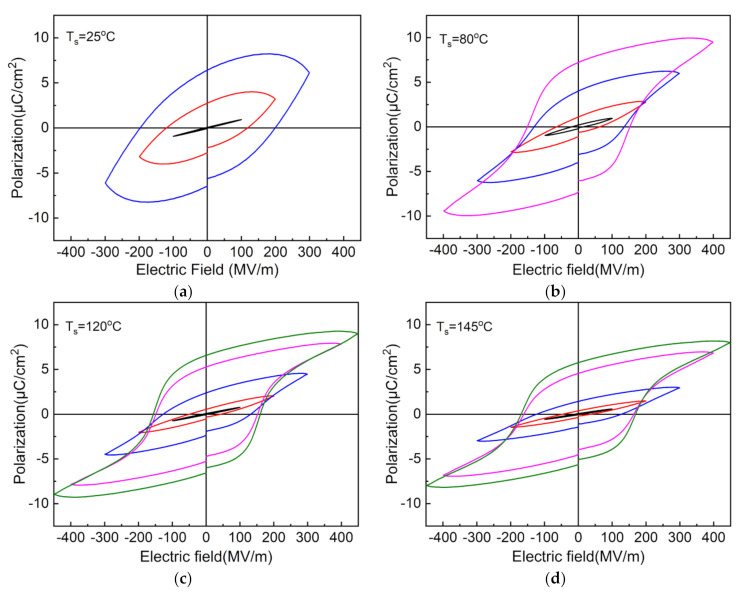
*D*-*E* hysteresis loops of the stretched nylon-11 films with stretching temperatures of (**a**) 25 °C, (**b**) 80 °C, (**c**) 120 °C and (**d**) 145 °C. The stretching ratio is set as 300%.

Figure 8 shows the *D*-*E* hysteresis loops of the 300% stretched nylon-11 films with different stretching temperatures. It should be noted that the nylon-11 film stretched at 25 °C can only withstand an electric field strength of more or less than 300 MV/m, while the ones stretched at the elevated temperatures can raise this value to 400 MV/m or more. The nylon-11 film stretched at 25 °C does not exhibit a typical ferroelectric hysteresis loop under its maximum electric field. In contrast, all the stretched films can demonstrate the ferroelectric hysteresis curves when the electric field is up to 300 MV/m. As the stretching temperature increases, the remanent polarization first increases from 6.4 to 7.2 μC/cm^2^ when the temperature is no more than 80 °C but then decreases to 5.7 μC/cm^2^ when the temperature exceeds 80 °C. The result suggests that stretching at a moderate temperature can facilitate the formation of the δ′ phase while an overly high stretching temperature may have an adverse effect on the generation of the δ′ phase, which is consistent with the previous discussion in Figure 4f. Besides, the coercive electric field first decreases from 199 MV/m to 153 MV/m when the stretching temperature is less than 80 °C but then rises to 170 MV/m when the stretching temperature continues to increase.

**Figure 9:**
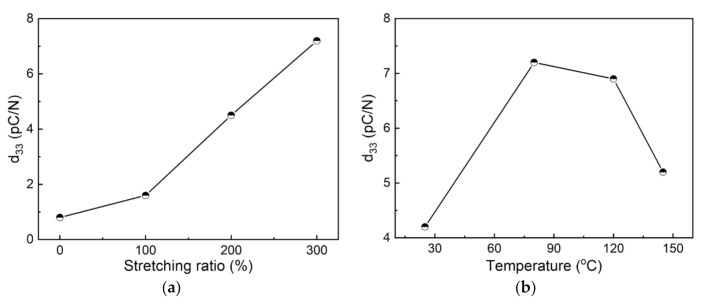
Piezoelectric coefficients (d_33_) of the nylon-11 films as a function of the (**a**) stretching ratio and (**b**) stretching temperature.

The piezoelectric coefficients (d_33_) of the unstretched and stretched nylon-11 films after thermal poling treatment were measured, as shown in Figure 9. Clearly, stretching causes a significant improvement of the d_33_ value. The unstretched nylon-11 film only possesses a small value of about 0.8 pC/N, while it rises to 7.2 pC/N as the SR increases to 300% (Figure 9a). Figure 9b shows the effect of the stretching temperature on the piezoelectric coefficient for the nylon-11 films. It is found that the d_33_ first increases from 4.2 to 7.2 pC/N and then decreases to 5.2 pC/N with the increase of the stretching temperature, achieving a maximum value at 80 °C. The excellent piezoelectric property acquired in the 300% nylon-11 film that stretched at 80 °C can be correlated well with its crystalline structure. According to the previous XRD results in Figure 2and, stretching can facilitate the formation of the δ’ phase; however, excessive temperatures of more than 80 °C would hinder the generation of the δ’ phase. Additionally, there is a strong relationship between the piezoelectric property and the content of the δ’ phase in nylon-11 [16,17]. As a result, the piezoelectric coefficient d_33_ can be significantly improved upon stretching but decreases when the stretching temperature exceeds 80 °C.

### 3.3. Performance of the nylon-11 based Piezoelectric Devices

**Figure 10:**
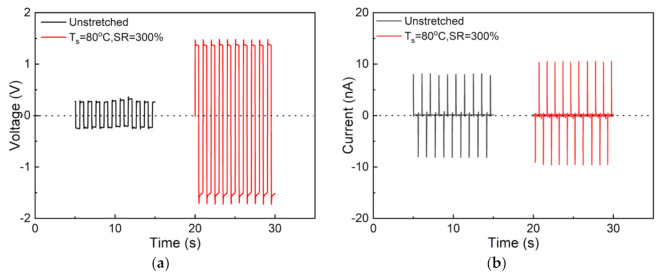
(**a**) Open-circuit voltage and (**b**) short-circuit current for the nylon-11 piezoelectric devices made of the unstretched and 300% stretched films (T_s_ = 80 °C) under a load of 50 N at 1 Hz.

The unstretched and 300% stretched nylon-11 films (T_s_ = 80 °C) were fabricated after thermal poling into piezoelectric devices in this work, and their performance was evaluated in terms of the output open-circuit voltage (V_oc_) and short-circuit current (I_sc_), as shown in Figure 10. It is found that both types of nylon-11-based piezoelectric devices can respond well to periodic external forces of 50 N and 1 Hz. The V_oc_ of the 300% stretched nylon-11 film is 1.5 V, which is about six times that of the unstretched one (0.25 V). Besides, the 300% stretched nylon-11 film also exhibits a higher I_sc_ (11 nA) than that of the unstretched one (8 nA). Comparatively, the improvement on the output current is not as much as that on the voltage. Similar results have been reported by other researchers, and they are ascribed to the huge internal resistance of the polymer-based device [32].

## 4. Conclusions

In this work, nylon-11 films were fabricated by the solution-casting method from a mixture of formic acid and dichloromethane and were then uniaxially stretched with different stretching ratios and temperatures. The two-dimensional wide-angle X-ray diffraction (2D-WAXD) results show that upon stretching, causes the γ phase to disappeared and leads to the generation of the δ′ phase in the nylon-11 films. The fraction of the δ’ crystal phase increased with the stretching ratio and acquired a maximum at an optimal stretching temperature of 80 °C, whereas a high stretching temperature of more than 120 °C had an adverse effect on the formation of the δ′ phase. The ferroelectric and piezoelectric properties of the stretched nylon-11 films could be correlated well with their crystalline structure. The optimum piezoelectric coefficient was 7.2 pC/N, which was obtained in the 300% stretched film at 80 °C, whose open-circuit voltage and short-circuit current were 1.5 V and 11 nA, respectively.

## Data Availability

Not applicable.
